# T lymphocyte senescence is attenuated in Parkinson’s disease

**DOI:** 10.1186/s12974-021-02287-9

**Published:** 2021-10-13

**Authors:** Antonina Kouli, Melanie Jensen, Vanesa Papastavrou, Kirsten M. Scott, Claire Kolenda, Craig Parker, Imtiaz H. Solim, Marta Camacho, Carmen Martin-Ruiz, Caroline H. Williams-Gray

**Affiliations:** 1 John Van Geest Centre for Brain Repair, Department of Clinical Neurosciences, University of Cambridge, Forvie Site, Robinson Way, Cambridge, CB2 0PY UK; 2 Department of Cellular Pathology, Charing Cross Hospital, Imperial College Healthcare NHS Trust, London, W6 8RF UK; 3 Bioscience Institute, BioScreening Core Facility, Campus for Ageing and Vitality, Newcastle University, Newcastle upon Tyne, NE4 5PL UK

**Keywords:** Parkinson’s disease, Immunosenescence, T lymphocytes, Ageing markers

## Abstract

**Background:**

Immune involvement is well-described in Parkinson’s disease (PD), including an adaptive T lymphocyte response. Given the increasing prevalence of Parkinson’s disease in older age, age-related dysregulation of T lymphocytes may be relevant in this disorder, and we have previously observed changes in age-associated CD8^+^ T cell subsets in mid-stage PD. This study aimed to further characterise T cell immunosenescence in newly diagnosed PD patients, including shifts in CD4^+^ and CD8^+^ subpopulations, and changes in markers of cellular ageing in CD8^+^ T lymphocytes.

**Methods:**

Peripheral blood mononuclear cells were extracted from the blood of 61 newly diagnosed PD patients and 63 age- and sex-matched controls. Flow cytometric analysis was used for immunophenotyping of CD8^+^ and CD4^+^ lymphocyte subsets, and analysis of recent thymic emigrant cells. Telomere length within CD8^+^ T lymphocytes was assessed, as well as the expression of the telomerase reverse transcriptase enzyme (hTERT), and the cell-ageing markers p16^INK4a^ and p21^CIP1/Waf1^.

**Results:**

The number of CD8^+^ TEMRA T cells was found to be significantly reduced in PD patients compared to controls. The expression of p16^INK4a^ in CD8^+^ lymphocytes was also lower in patients versus controls. Chronic latent CMV infection was associated with increased senescent CD8^+^ lymphocytes in healthy controls, but this shift was less apparent in PD patients.

**Conclusions:**

Taken together, our data demonstrate a reduction in CD8^+^ T cell replicative senescence which is present at the earliest stages of Parkinson’s disease.

**Supplementary Information:**

The online version contains supplementary material available at 10.1186/s12974-021-02287-9.

## Introduction

It is well-recognised that numerous innate and adaptive immune changes occur both in the brain and the periphery in Parkinson’s disease (PD) patients [1], though the precise mechanisms and their role in disease progression are still poorly understood. A better understanding of the immune pathways involved in PD pathogenesis is needed, both to identify predictive biomarkers and to identify rational therapeutic targets for disease-modification.

Age-related immune changes (immunosenescence) may be particularly relevant to diseases like PD for which ageing is a major risk factor. Immunosenescence leads to increased infection susceptibility and reduced effectiveness of vaccines [2]. This is mediated primarily by changes in CD8^+^ T lymphocytes. A critical phenotypic change in these cells is the loss of surface expression of CD28 and upregulation of CD57 [3–5]. Functionally, “senescent” CD28^lo^CD57^hi^ lymphocytes have limited proliferative potential, decreased cytolytic function, and are clonally restricted. Immunosenescence is also characterised by a decrease in naïve cells coupled with expansion of late-differentiated antigen-exposed cells [6]. Terminally differentiated effector memory cells re-expressing CD45RA (TEMRA)—replicative senescent cells with diminished proliferative ability—are a marker of age-associated immune dysregulation [7]. CD8^+^ T cell senescence is strongly associated with exposure to common latent viral infections, such as cytomegalovirus (CMV) and Epstein–Barr virus (EBV), with increases in both antigen-specific CD8^+^ CD28^lo^CD57^hi^ and CD8^+^ TEMRA populations reported in seropositive populations [2, 8, 9]. Similar age-related changes may also occur in the CD4^+^ T cell compartment [10].

Atrophy of the thymus gland, a key source of clonally diverse naïve T lymphocytes, is another key feature of immunosenescence. Thymic decline with age limits the naïve T cell repertoire, with consequent impaired recognition of novel antigens [11, 12]. Thymic function can be quantified non-invasively by the measurement of recent thymic emigrants (RTEs) in the peripheral blood [13]. CD103^+^ and PTK7^+^ naïve cells within the CD8^+^ and CD4^+^ pool, respectively, have been identified as key markers of RTEs [14, 15]. Telomeres, the protective nucleoprotein structures located at the ends of chromosomes, shorten with age, and have been proposed as a biomarker of cell ageing [16]. Telomere shortening and reduction of telomerase activity (a critical counter to telomere attrition) occur in senescent T cells [17]. Expression of hTERT, a rate-limiting catalytic subunit of telomerase, also progressively declines during T cell differentiation [18]. Cellular senescence is also associated with an increase in the cyclin-dependent kinase inhibitors p16^INK4a^ and p21^CIP1/Waf1^, and their gene expression levels within lymphocytes have been explored as biomarkers in age-related cardiac and lung diseases [19, 20].

Investigation of immunosenescence markers in PD has been limited to date. Our previous study is the only one to our knowledge which has specifically investigated senescent T cell subsets in PD patients. Using flow cytometric analysis of peripheral blood mononuclear cells (PBMCs), we observed a more activated and less senescent CD8^+^ T cell profile in PD cases (*n* = 41) compared to age and sex-matched controls (*n* = 41), with a reduced percentage of CD28^lo^CD57^hi^ cells and TEMRA cells [21]. Our findings support the proposed role of immune hyperactivity and inflammation in PD initiation and progression [22, 23]. Interestingly, CMV positivity was associated with CD8 senescence (as expected), in controls, but not in PD patients, raising the possibility that PD might be associated with intrinsic differences in the CD8^+^ response to viral infection. We found no significant changes in markers of CD4^+^ replicative senescence in PD versus controls, but this warrants replication given the evidence for altered CD4^+^ T cell subsets in PD [21, 24–26]. Thymic output in PD has not previously been explored.

Prior studies exploring telomere length in PD are equivocal: a recent meta-analysis of eight studies showed no difference between 956 PD patients and 1284 controls when pooling across different tissue types [27]. However, longer telomere length in peripheral blood mononuclear cells of PD patients versus controls has been observed. [28]. In addition, longer leukocyte telomeres have been identified as a risk factor for developing dementia within 3 years of PD diagnosis [29], although our own prior study had opposing findings, with shorter telomeres in incident PD cases who went on to develop a dementia within 3 years [30]. We also found that leukocyte expression levels of the cellular senescence marker p21 were lower in PD versus controls, whilst lower p16 expression at baseline was associated with faster motor and cognitive progression over 36 months; thus, supporting the hypothesis that immunosenescence is reduced in PD.

On the basis of our previous findings, we hypothesise that attenuated T cell senescence is relevant in PD and warrants further investigation as a potential biomarker of the disease. This current study aimed to validate our previous findings with respect to alterations in CD8^+^ senescent cells in PD, as well as to comprehensively characterise T cell immunosenescence, evidence of prior CMV and EBV infection, thymic emigrants, telomere length, and expression of telomerase (hTERT), p16 and p21. We examined a newly diagnosed PD cohort to determine whether T cell senescence markers are altered in the earliest stages of the disease and evaluated relationships with clinical disease characteristics and predicted clinical outcomes.

## Materials and methods

### Participants

Recently diagnosed PD patients (≤ 2 years from diagnosis, Hoehn & Yahr ≤ 2) fulfilling UK PD Brain Bank Criteria were recruited from the Parkinson’s Disease Research Clinic at the John Van Geest Centre for Brain Repair in Cambridge. Controls were recruited from the NIHR Cambridge Bioresource (cambridgebioresource.org.uk) or from the Parkinson’s Disease Research Clinic and were age-, and sex-matched to patients. Exclusion criteria for both patient and control groups were the presence of chronic inflammatory or autoimmune disorders, current or latent infection, vaccinations in the preceding month and use of anti-inflammatory/immune-modulating medications. Additional exclusion criteria for PD patients were diagnosis of dementia according to MDS PD-dementia criteria and the presence of significant psychiatric disturbances. Controls had no history of neurological disease or depression and no self-reported memory problems.

All participants were clinically assessed for comorbid conditions, and medication history. PD cases underwent standardised assessments of motor and cognitive dysfunction and mood, including the MDS-Unified Parkinson's Disease Rating Scale (MDS-UPDRS, completed in the ‘on’ medication state), and Addenbrooke’s cognitive examination (ACE-III). Levodopa equivalent daily doses were calculated (adapted from [31]. Predicted outcome at 5 years (development of postural instability, dementia, death) was calculated based on baseline clinical variables (age, UPDRS axial score and semantic fluency) using a validated prognostic model [32]. Ethical approval was obtained from the East of England—Essex Research Ethics Committee (16/EE/0445) and the East of England—Cambridge Central Research Ethics Committee (03/303).

### Sample collection and PBMC extraction

In total, 50 ml of venous blood were collected using S-Monovette® tubes (Sarstedt): lithium heparin for peripheral blood mononuclear cell (PBMC) isolation, ethylenediaminetetraacetic acid (EDTA) for full blood count, and clot activator tubes for separation of serum. Full blood count analysis was done in the pathology laboratories at Addenbrooke’s hospital, Cambridge. Samples for serum extraction were left for 15 min to clot prior to centrifugation at 2000 rpm for 15 min. Serum was analysed in the pathology laboratories at Addenbrooke’s hospital, Cambridge for C-reactive protein (CRP), cytomegalovirus (CMV) IgG and Epstein–Barr virus (EBV) IgG.

All blood samples were processed immediately after collection. PBMCs were isolated from whole blood via standard density gradient using Ficoll (GE Healthcare). After extraction, the PBMCs were counted on a haemocytometer and divided for immunophenotyping assays and for isolation of CD8^+^ T cells to carry out telomere length measurement and gene expression analysis.

### Flow cytometry immunophenotyping

PBMCs were plated in a clear 96-well plate at 1 × 10^6^ cells/well. After blocking for 30 min at 4 °C in FACS buffer (0.1% BSA and 0.01% sodium azide in PBS) containing 2% mouse serum, the samples were incubated with the appropriate antibodies. The T cell senescence panel included the following: CD3 (APC; BD Biosciences), CD4 (BV510; BD Biosciences), CD8 (APC H7; BD Biosciences), CCR7 (FITC; BD Biosciences), CD45RA (PE; Biolegend), CD28 (PE Cy7; BD Biosciences), and CD57 (PerCP Cy5.5; Biolegend). Recent thymic emigrant (RTE) phenotyping was done in a subset of PD patients and controls. The RTE panel included the following: CD3 (APC; BD Biosciences), CD4 (BUV395; Biolegend), CD8 (APC H7; Biolegend), CD45RA (PerCP Cy5.5; BD Biosciences), CD103 (FITC; BD Biosciences), and PTK7 (PE; BD Biosciences). After antibody incubation, the samples were washed twice with FACS buffer and fixed with 2% paraformaldehyde. Post-fixation, the cells were washed twice and were transferred to FACS tubes.

Flow cytometry was run within 24 h of PBMC extraction. Data were acquired on a BD LSR Fortessa™ flow cytometer using the BD FACS Diva software at the NIHR Cambridge BRC Cell Phenotyping Hub. Single-stained samples were used for each experiment and compensation was applied to correct for fluorescence spectral overlap. Isotype controls were used where appropriate to determine non-specific antibody binding. Fluorescence minus-one controls were used to check the gating strategy for cell populations of interest.

### Data analysis

Flow cytometry data analysis was done using FlowJo v10 software (BD Life Sciences). Lymphocytes were gated using forward and side scatter area and the single cells were selected on a forward scatter area versus height plot. CD4 and CD8 T lymphocytes were gated as CD3^+^CD4^+^ and CD3^+^CD8^+^ cells, respectively. Quadrant gating of CCR7 versus CD45RA plots was used to gate naïve, central memory, effector memory and T effector memory cells that re-expresses CD45RA (TEMRA) within both CD4^+^ and CD8^+^ populations. Within the CD8^+^ T cell population, senescent “late differentiated” cells were also defined as CD28 low and CD57 high (CD28^lo^CD57^hi^), in addition to the TEMRA subset. Data are presented both as % of total lymphocytes and as absolute counts (calculated based on the full blood counts). One PD sample has been removed from the flow cytometry dataset prior to statistical comparison of groups due to a technical issue during the cytometry experiment. In the recent thymic emigrant panel, CD4^+^ RTEs were defined as CD45RA^+^ PTK7^+^ cells, and CD8^+^ RTEs were defined as CD45RA^+^ CD103^+^ cells.

### CD8 T cell separation

CD8^+^ T cell subsets were separated by magnetic cell sorting using MACS® CD8 magnetic beads (Miltenyi Biotec), as per the manufacturer’s instructions. Briefly, freshly extracted PBMCs were resuspended in MACS buffer (5 mg/ml BSA and 2 mM EDTA in PBS), then incubated with CD8 magnetic bead suspension for 15 min at 4 °C. After washing and resuspending in MACS buffer, the cell suspension was added to pre-rinsed LS separation columns placed in a MidiMACST^M^ magnet separator (Miltenyi Biotec). After the negative fraction had run through, the column was rinsed 3 times with MACS buffer. Finally, the column was removed from the magnet and CD8^+^ cells were eluted with 5 ml MACS buffer. The separated cells were stored at −80 °C until analysis.

### Telomere length analysis

Telomere length was measured using a previously published quantitative PCR (qPCR) method with modifications [33]. All samples were assessed in triplicate and all PCRs were carried out on a QuantStudio™ 7 Flex Real-Time PCR System with 384-well plate capacity (Applied Biosystems). Telomere length measurements are expressed as a ratio of telomere base pairs/single copy gene base pairs (T/S ratio). Three internal control DNA samples of known telomere length (10.4 kb, 3.9 kb, and 2 kb) were run within each plate to correct for plate-to-plate variation. As a further authentication of our telomere length measurements, we performed a revaluation of those samples that were in either the top or bottom 5% of the telomere length distribution as well as those samples that gave no valid data on the first run. The intra-assay coefficient of variation was 4.04% while the inter-assay coefficient of variation was 1.27%.

### Gene expression of hTERT, p16 and p21

The quantification of expression levels of hTERT, p16 and p21 was performed by RT-qPCR analysis on a QuantStudio™ 7 Flex Real-Time PCR System with 384-well plate capacity (Applied Biosystems), using PGK1 as reference gene and an internal RNA control sample. Briefly, 500 ng of RNA was converted into cDNA by means of qScript cDNA synthesis kit (Quanta Biosciences, Beverley, MA, USA) on a 20 μl reaction that included 1 μl of qScript reverse transcriptase. The reaction comprised 5 min at 22 °C, 30 min at 42 °C and 5 min at 85 °C. This was followed by a 15 μl pre-amplification reaction by means of PrimePCR^TM^PreAmp primers for hTERT, p16, p21, and PGK1 (BioRad, Watford, UK) with SsoAdvanced Preamp Supermix (BioRad, Watford, UK) under the following conditions: 3 min at 95 °C; 12 cycles of 15 s at 95 °C plus 4 min at 58 °C; and 5 min at 12ºC. The resulting pre-amplified samples where then diluted 1:5 and applied onto a 10 μl qPCR reaction. The following PrimePCR™ SYBR® primer sets (BioRad, Watford, UK) were used: qHsaCED0056722 (p16); qHsaCID0014498 (p21); qHsaCID0009247 (hTERT) and qHsaCED0042912 (PGK1); the reactions included 1.5 μl of pre-amplified cDNA and 5 μl of Applied Biosystems Power SYBR™ Green PCR Master Mix (ThermoFisher Scientific, Waltham, MA, USA). All four qPCR reactions (hTERT, p16, p21, and PGK1) were performed in duplicate, with all samples run on the same plate. The thermo-cycling programme included 2 min at 50 ºC; 10 min at 95 °C and 40 cycles of 15 s at 95 °C and 30 s at 60 °C as well as a melting curve analysis. Values of expression levels for hTERT, p16 and p21 were calculated by ΔCΤ based on the differences on threshold cycle for hTERT, p16 and p21 against PGK1 and relative gene expression was calculated by the formula 2^–ΔΔCT^ based on ΔCΤ values for the samples against those obtained for the internal control. Additional information on the primers used is provided in Additional file 1: Table S1.

### Statistical analysis

Comparisons between PD and controls were performed with two-tailed unpaired t-tests, and comparisons between multiple groups were done using two-way ANOVA with Sidak’s post hoc test correcting for multiple comparisons. Categorical variables were compared using Fisher’s exact test. Pearson product–moment correlation was used to assess correlations between cell markers and clinical variables. The data are presented as the mean (SD). A *p*-value < 0.05 was defined as statistically significant. SPSS (IBM SPSS Statistics for Windows, Version 22.0) and GraphPad Prism (GraphPad Software, Version 6.04 for Windows) were used for statistical analyses. Graphs were generated using R.

## Results

### Demographics

61 PD patients and 63 controls were recruited. Their demographics are summarised in Table 1. The two groups were well-matched for age (*p* = 0.957) and sex (*p* = 0.105), as well as CMV and EBV seropositivity (*p* = 0.719 and *p* = 0.267, respectively). All patients had early-stage PD (0.97 ± 0.54 years disease duration). PD patients had lower cognitive scores compared to the controls as measured by ACE-III (*p* = 0.011). C-reactive protein (CRP) was within the normal range (< 4 mg/L) in all participants, thus excluding concurrent infections at the time of blood sampling.

**Table 1:** PD patient and control demographics

	Control	PD	*p*
Sample size	63	61	–
Sex (% male)	51%	66%	0.105
Age at visit	67.5 (7.2)	67.4 (7.1)	0.957
CMV (% positive)	44%	48%	0.719
EBV (% positive)	92%	85%	0.267
ACE-III	94.1 (7.4)	93.0 (4.5)	0.011*
Disease duration (years)	–	0.97 (0.54)	–
MDS-UPDRS-III (motor score)	–	27.8 (10.3)	–
MDS-UPDRS total	–	47.4 (15.1)	–
Levodopa equivalent daily-dose (LEDD)	–	290 (163.5)	–

CMV, Cytomegalovirus; EBV, Epstein–Barr virus; ACE-III, Addenbrooke’s cognitive examination III; MDS-UPDRS, Movement Disorder Society—Unified Parkinson’s Disease Rating Scale (measured on medication). ACE-III was done in all PD patients and a subset of 41 controls. The values represent the mean (SD); *p < 0.05

### Reduction in total lymphocytes, cytotoxic CD8^+^ T cells and CD8^+^ TEMRA cells in PD patients compared to controls

Full blood count analysis revealed a significant reduction in the total number of lymphocytes in PD patients compared to healthy controls (*p* = 0.043), as has been previously reported [34]. The absolute count of cytotoxic CD8^+^ T lymphocytes was also reduced in patients versus controls (*p* = 0.018) (Table 2).

**Table 2:** Total lymphocytes and main T cell subsets

	% of total lymphocytes			Absolute cell count (cells × 10^9^/L)		
	Control	PD	*p*	Control	PD	*p*
Total	–	–	–	1.553 (0.47)	1.397 (0.37)	0.043*
CD4^+^	47.41 (12.02)	50.88 (11.95)	0.113	0.736 (0.31)	0.696 (0.22)	0.410
CD8^+^	23.72 (11.48)	20.88 (8.86)	0.126	0.380 (0.26)	0.292 (0.14)	0.018*

Comparisons were done using two-tailed unpaired t-tests. The values represent the mean (SD); *p < 0.05. Significant values are in italics

**Fig. 1:**
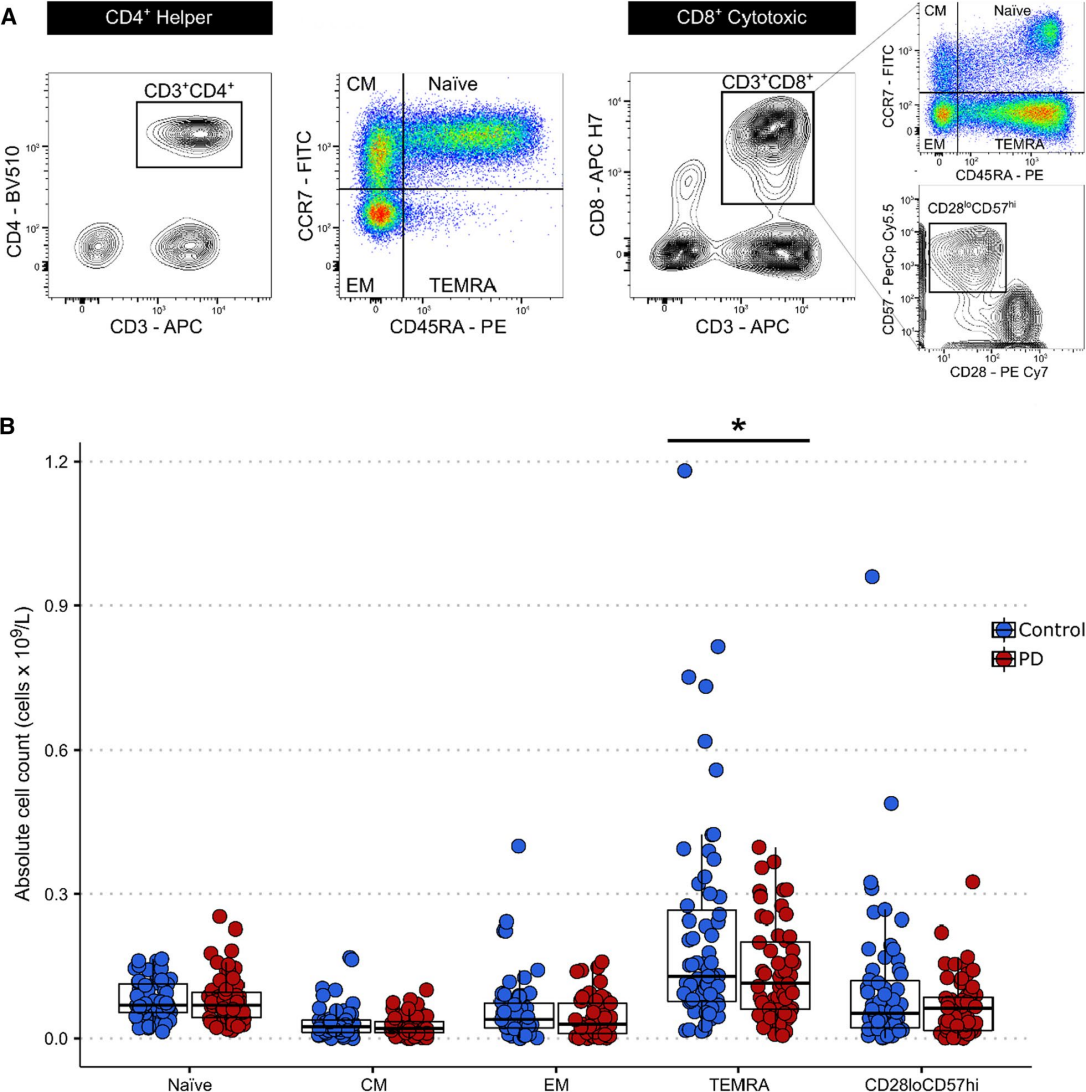
T lymphocyte immunophenotyping in PD patients versus controls. A Following gating of the total lymphocyte and single cell populations, CD4^+^ and CD8^+^ T cells were identified as CD3^+^CD4^+^ and CD3^+^CD8^+^, respectively. CD4^+^ and CD8^+^ subsets were distiguished using a quadrant gate based on the expression of CCR7 and CD45RA. Additionally, senescent CD8^+^ subsets were identified based on their low expression of CD28 and high expression of CD57. B Quantification of CD8^+^ T cell subets in PD patients and controls. There was a significant decrease in the absolute number of CD8^+^ TEMRA cells in patients versus controls (two-tailed unpaired Welch's t-test, t(87.25) = 2.392, *p* = 0.019). All other CD8^+^ T cell subsets did not differ between the groups (two-tailed unpaired Welch's t-test, *p* > 0.05). CM: Central memory, EM: effector memory, TEMRA: terminally differentiated effector memory cells re-expressing CD45RA. *p < 0.05

Flow cytometry was used to measure the proportions of relevant T cell subsets within the lymphocyte population. Based on these percentages and the total lymphocyte counts for each sample, the absolute number of cell subsets was calculated (Table 3, Fig. 1). All subsequent results refer to absolute counts only. There was a significant reduction in the absolute number of CD8^+^ TEMRA cells in patients compared to controls (*p* = 0.019). A similar trend was observed in the number of senescent CD8^+^ CD28^lo^CD57^hi^ cells, though this did not reach significance (*p* = 0.087) (Fig. 1B). CD8^+^ TEMRA and CD8^+^ CD28^lo^57^hi^ cell counts were closely correlated in PD patients (Pearson r = 0.766, *p* < 0.001) as well as in controls (Pearson *r* = 0.769, *p* < 0.001). There were no differences in CD4^+^ subsets between PD patients and controls.

**Table 3:** CD4^+^ and CD8^+^ T lymphocyte subsets

	% of total lymphocytes			Absolute cell count—cells × 10^9^/L		
	Control	PD	*p*	Control	PD	*p*
CD4^+^						
Naïve	24.9 (14.4)	28.2 (14.6)	0.210	0.388 (0.282)	0.374 (0.183)	0.761
Central memory	14.4 (7.3)	15.1 (7.5)	0.624	0.217 (0.115)	0.214 (0.125)	0.899
Effector memory	6.0 (4.0)	5.7 (3.7)	0.656	0.095 (0.069)	0.082 (0.069)	0.332
TEMRA	2.1 (3.5)	1.9 (2.4)	0.734	0.037 (0.072)	0.025 (0.032)	0.246
CD8^+^						
Naïve	5.3 (2.7)	5.9 (3.2)	0.313	0.080 (0.040)	0.081 (0.050)	0.967
Central memory	2.2 (2.1)	1.9 (1.3)	0.277	0.033 (0.033)	0.027 (0.021)	0.205
Effector memory	3.6 (3.7)	2.9 (3.6)	0.223	0.058 (0.068)	0.043 (0.041)	0.131
TEMRA	12.6 (9.7)	10.2 (7.0)	0.121	0.213 (0.221)	0.140 (0.100)	0.019*
CD28^lo^CD57^hi^	5.9 (6.5)	4.9 (4.5)	0.327	0.100 (0.145)	0.066 (0.061)	0.087

TEMRA, terminally differentiated effector memory cells re-expressing CD45RA. Comparisons were done using two-tailed unpaired t-tests. The values represent the mean (SD); *p < 0.05. Significant values are in italics

Amongst the PD cases, there were no significant correlations between the CD8^+^ TEMRA or CD28^lo^CD57^hi^ senescent T cell counts and clinical variables, including age, sex, measures of motor and cognitive function, 5-year prognostic score or LEDD.

### *The association between prior CMV infection and CD8*^+^*T cell senescence is attenuated in PD*

**Fig. 2:**
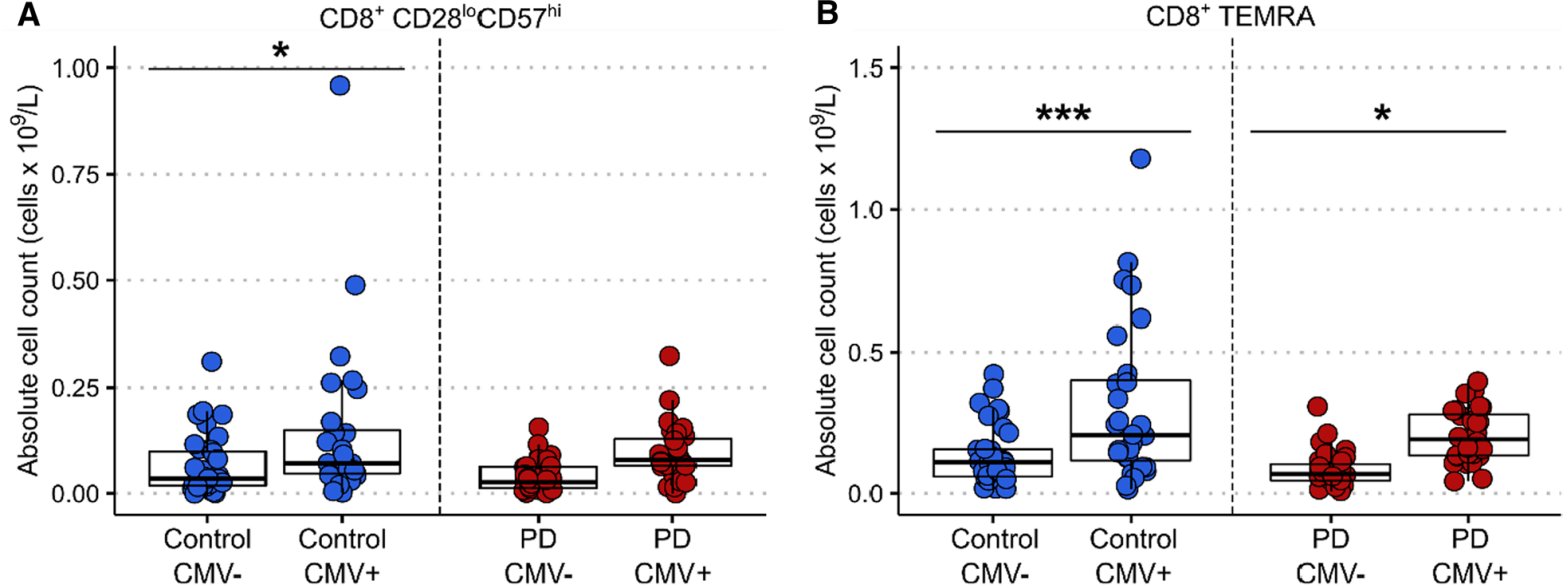
Senescent CD8^+^ lymphocytes in CMV-positive versus CMV-negative patients and controls. **A** Two-way ANOVA with Sidak’s multiple comparisons test showed a statistically significant main effect of CMV seropositivity, F(1,118) = 11.9, *p* < 0.001. Post hoc pairwise comparison revealed a significant increase in the absolute count of CD8^+^ CD28^lo^CD57^hi^ T lymphocytes in CMV-positive compared to CMV-negative controls (*p* = 0.026). There was no statistically significant difference between CMV-positive and CMV-negative PD patients (*p* = 0.260). **B** Two-way ANOVA with Sidak's multiple comparisons test showed a statistically significant main effect of CMV seropositivity F(1,118) = 26.16, *p* < 0.0001 and main effect of group F(1,118) = 7.382, *p* = 0.008. Post hoc pairwise comparison revealed a significant increase in the absolute count of CD8^+^ TEMRA cells in CMV-positive compared to CMV-negative controls (*p* < 0.001) and in CMV-positive compared to CMV-negative PD patients (*p* = 0.026) CMV: Cytomegalovirus, TEMRA: terminally differentiated effector memory cells re-expressing CD45RA. ***p < 0.001,*p < 0.05

There was no significant difference in either CMV or EBV seropositivity between PD patients and controls (*p* = 0.719 and *p* = 0.267, respectively) (Table 1). Comparison of CD8^+^ CD28^lo^CD57^hi^ T lymphocytes revealed a significant increase in their absolute number (*p* = 0.026) in CMV-positive versus CMV-negative controls (Fig. 2A). In contrast, in the patient group, the CD28^lo^CD57^hi^ T cell count did not differ between CMV-positive versus CMV-negative cases (*p* = 0.260). CD8^+^ TEMRA cells were significantly higher in CMV-positive versus CMV-negative cases in both the control and PD group, but the effect was more significant in the controls (*p* < 0.001) compared to the patients (*p* = 0.026) (Fig. 2B). There were no differences in the absolute counts of CD8^+^ TEMRA or CD28^lo^CD57^hi^ T cells in EBV-positive or EBV-negative patients and controls (data not shown).

### No evidence of changes in thymic emigration of T cells in PD versus controls

**Fig. 3:**
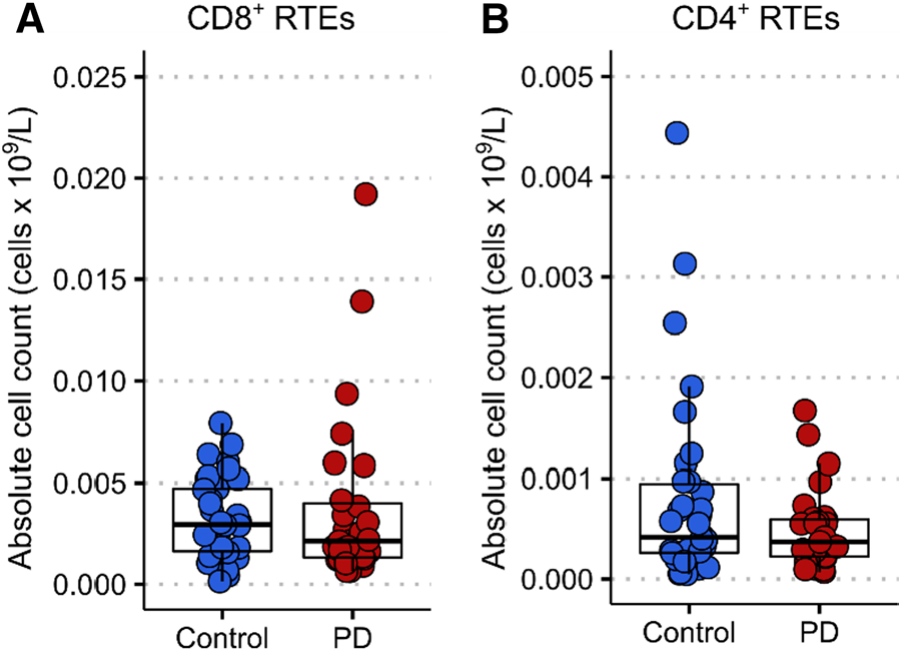
CD8^+^ and CD4^+^ recent thymic emigrant (RTE) lympocytes in a subet of PD patients versus controls. **A** The absolute number of CD8^+^ RTEs did not differ between PD patients and controls (two-tailed unpaired Welch’s t-test, t(34.86) = 0.662, *p* = 0.512). **B** The absolute number of CD4^+^ RTEs did not differ between PD patients and controls (two-tailed unpaired Welch’s t-test, t(46.44) = 1.625, *p* = 0.111). Control *n* = 34, PD *n* = 27

Recent thymic emigrants (RTEs) were investigated in a subset of PD patients (*n* = 27) and controls (*n* = 34). The number of either CD8^+^ (CD45RA^+^CD103^+^) or CD4^+^ (CD45RA^+^PTK7^+^) RTEs was not significantly different in PD patients compared to controls (*p* = 0.512 and *p* = 0.111, respectively) (Fig. 3A, B). Amongst the PD cases, there were no significant correlations between the CD4^+^ or CD8^+^ RTEs counts and age or sex.

### Expression of the cell aging marker p16 is reduced in PD compared to controls

**Fig. 4:**
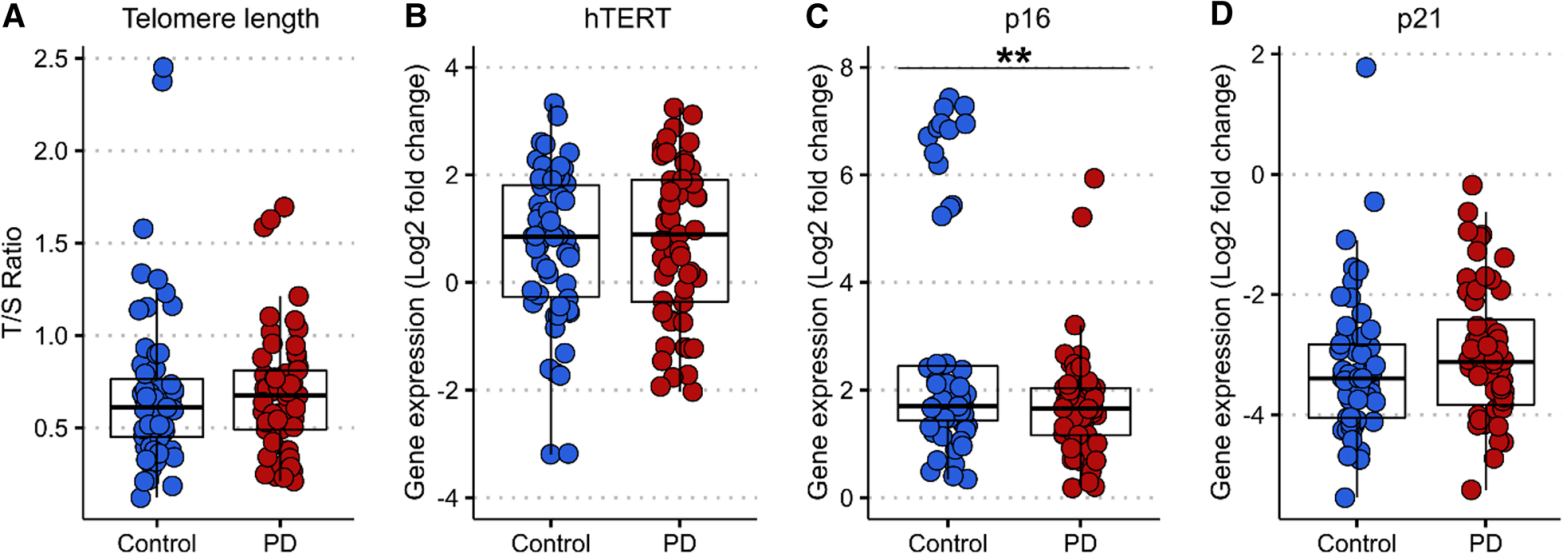
Telomere length and cell-ageing markers in CD8^+^ lymphocytes of PD patients compared to controls. **A** Telomere length is expressed as a ratio of telomere base pairs/single copy gene base pairs (T/S ratio), efficiency corrected [35]. Two-tailed unpaired Welch’s t-test, t(108.2) = 0.245, *p* = 0.807. **B** Relative mRNA expression levels of hTERT expressed as the log2-fold change (2^−∆∆CT^). Two-tailed unpaired Welch’s t-test, t(62.76) = 1.159, *p* = 0.251. **C** Relative mRNA expression levels of p16 expressed as the log2-fold change (2^−∆∆CT^). Two-tailed unpaired Welch’s t-test, t(62.74) = 3.274, *p* = 0.002. **D** Relative mRNA expression levels of p21 expressed as the log2-fold change (2^−∆∆CT^). Two-tailed unpaired Welch’s t-test, t(74.73) = 1.143, *p* = 0.886. hTERT: human telomerase enzyme reverse transcriptase. Control *n* = 59, PD *n* = 57. **p < 0.001

Given that our data confirmed alterations in senescent cell subtypes within the CD8^+^ population, CD8^+^ lymphocytes were isolated for further experiments to assess cell-ageing markers. Telomere length, as well as the expression of the telomerase reverse transcriptase enzyme (hTERT) in CD8^+^ T lymphocytes were similar in PD and control groups (*p* = 0.807 and *p* = 0.251, respectively) (Fig. 4A, B). There was a significant decrease in the expression level of the cell-ageing marker p16 in CD8^+^ T cells of PD patients versus controls (*p* = 0.002) (Fig. 4C). No differences were observed in p21 gene expression between groups (*p* = 0.886) (Fig. 4D). Telomere length as well as hTERT, p16 and p21 expression did not correlate with clinical data including age, sex, disease duration, UPDRS-III, ACE-III and LEDD.

p16 expression appears to have a biphasic distribution in our dataset, with a larger proportion of controls in the ‘high’ cluster (Fig. 4C). This group of controls did not differ significantly from the control population as a whole in terms of age or sex.

## Discussion

This study has comprehensively investigated markers of T cell senescence in a newly diagnosed PD cohort and age-matched controls, and provides evidence of a reduction in CD8^+^ TEMRA cells, as well as lower expression of the cell-ageing marker p16 in CD8^+^ T lymphocytes in PD. This validates and extends our previous findings [21], demonstrating that reduced CD8^+^ cell senescence is a feature of early PD. The current cohort comprised patients with a mean disease duration of 0.97 (0.54) years in contrast to 4.3 (1.2) years in our previous study. Although we have demonstrated that changes in CD8^+^ cell senescence markers are present from early on in the disease course, our data do not support their use as diagnostic biomarkers, given the significant overlap between PD patients and controls. Nonetheless, our observations provide an insight into early T lymphocyte changes in PD, with important implications for better understanding of the immune basis of this condition.

With respect to changes in T cell subsets, our main finding was a reduction in CD8^+^ TEMRA cells. We observed similar findings in our previous study [21], as well as a reduction in CD8^+^ CD28^lo^CD57^hi^ lymphocytes. There was a trend towards reduction in CD8^+^ CD28^lo^CD57^hi^ cells in PD cases in this study which did not reach significance, but CD8^+^ TEMRA and CD8^+^ CD28^lo^CD57^hi^ cell counts were strongly correlated and are likely to represent overlapping populations [6]. The difference in CD8^+^ senescent subsets between groups was not driven by differences in age or prior infection with CMV or EBV. However, our data suggest a reduced impact of CMV seropositivity on CD8^+^ senescence in patients versus controls. Prior CMV infection was associated with elevated CD8^+^ CD28^lo^CD57^hi^ cells in controls but not PD cases, which is in keeping with our previous findings [21]. CMV seropositivity was also associated with increased CD8^+^ TEMRA cells in controls, and although a similar effect was seen in PD cases, this was less significant. CMV infection is thought to be a critical driver of CD57 expression in CD8^+^ T lymphocytes in older individuals [36, 37]. In two independent cohorts, we have observed that this relationship between prior CMV infection and CD8^+^ CD28^lo^CD57^hi^ cells is reduced in PD. This raises the intriguing possibility that some people who develop PD might have intrinsic differences in CD8^+^ immune function, with an attenuated accumulation of CD8^+^ CD28^lo^CD57^hi^ cells upon chronic exposure to viruses.

We also interrogated whether the reduction in T cell senescence in PD was associated with relative preservation of thymic function, which would be expected to decline with age [11]. However, our findings show no differences in the number of RTEs between PD patients and controls, suggesting that thymic function does not contribute to alterations in the balance of naïve versus senescent cells in PD.

Replicative cell senescence has been associated with the gradual shortening of telomeres [38], and leukocyte telomere length has been implicated as biomarker in PD, although with inconsistent findings across studies [27–30]. We measured telomere length and expression of hTERT within the CD8^+^ lymphocyte population and did not observe any significant differences in the length of telomeres, nor in hTERT expression in PD versus controls.

The cyclin-dependent kinase inhibitors p16^INK4a^ and p21^CIP1/Waf1^ are both well-established biomarkers of cell senescence [39, 40]. Blood-based expression of these markers has not been well-studied in the context of neurodegeneration, although p21 expression in monocytes has been reported to be lower in Alzheimer’s disease [41], and we have previously found evidence of reduced p21 expression in total leukocytes in early PD, as well as an association between lower leukocyte p16 expression and more rapid disease progression [30]. In the current study, where we focused our investigation on the CD8^+^ T cell subset, we found no difference in p21 expression, but reduced p16 expression in PD patients versus controls, providing further evidence of an attenuation of CD8^+^ T cell senescence in PD.

One interpretation of our findings is that the observed reduction in senescent CD8^+^ T lymphocytes reflects a pre-existing intrinsic difference in the adaptive immune system in individuals who develop PD, perhaps due to an altered response to earlier viral infections, or due to reduced survival of the CD8^+^ TEMRA population. The shift of CD8^+^ T cells towards a senescent phenotype in response to viral exposure during normal ageing may have a protective effect in terms of PD risk, through limiting immune activation to novel or disease-associated antigens and consequently reducing neuroinflammation. This is in line with data showing that CMV infection is not associated with all-cause or cardiovascular mortality in older adults [42]. In PD, a more active and less senescent T cell profile may be responsible for driving an exaggerated response to PD-associated antigens, such as α-synuclein. Indeed, recent evidence from human studies suggests that α-synuclein epitopes are recognised by autoreactive T lymphocytes in PD [43].

Alternatively, it is possible that the significance of CD8^+^ TEMRAs relates to their cytotoxic capacity. Although TEMRAs are late-differentiated lymphocytes with characteristics of replicative senescence, they remain highly cytotoxic on stimulation by their target antigen and may play a critical role in certain disease states, for example driving graft failure in renal transplant patients [44]. CD8^+^ TEMRAs with specificity for disease relevant antigens could be sequestered out of the blood and into the central nervous system (CNS) in PD, where they may drive a neurotoxic response. Post-mortem studies have shown increased infiltration of both CD4^+^ and CD8^+^ T lymphocytes into the PD brain [45–47]. It is therefore possible that the reduction of highly cytotoxic CD8^+^ TEMRA cells we observed in the PD blood might be due to their migration into the CNS and infiltration into the brain parenchyma. Immunophenotyping of T lymphocytes in the cerebrospinal fluid (CSF) in PD has been limited to date, and to our knowledge, neither CD8^+^ TEMRA cells nor CD8^+^ CD28^lo^CD57^hi^ cells have been studied. However, a recent study in Alzheimer’s disease reported an increase in clonally expanded CD8^+^ TEMRAs in the CSF of patients versus controls. In contrast to our findings, they also reported increased CD8^+^ TEMRA cells (% of total PBMCs) in the blood [48]. However, the TEMRA population was defined differently (CD8^+^CD45RA^+^CD27^−^, as opposed to CD8^+^CD45RA^+^CCR7^−^); furthermore, T cell immunosenescence may play differing roles in Alzheimer’s and Parkinson’s diseases.

An important limitation of our study is that we did not perform functional characterisation of CD8^+^ T cell subsets to demonstrate their proliferative versus senescent capacity. It would also be of interest to determine the clonal specificity of CD8^+^ senescent subtypes in PD.

## Conclusion

In conclusion, our data show a reduction in CD8^+^ T lymphocyte senescence in newly diagnosed PD patients compared to age- and sex-matched controls. This is evidenced by a decrease in the CD8^+^ TEMRA subpopulation and downregulation of the senescence biomarker p16. The reduction in senescent T cells was not related to a lower incidence of prior infection with viral pathogens such as CMV or EBV in PD cases, but rather our data indicate that the typical viral-induced senescent shift in the CD8^+^ population may be attenuated in PD. The alterations in CD8^+^ lymphocytes were present in early disease and were not clearly associated with markers of disease severity, suggesting that they may have relevance to PD onset rather than progression. Further investigation of markers of T cell senescence in prodromal and longitudinal PD cohorts is warranted, along with functional characterisation of these senescent cell subtypes and measurement of their infiltration into the CNS.

## Supplementary Information

**Additional file 1: Table S1.** List of qPCR primers.

## Data Availability

Supporting data related to the findings of this study will be made available by the authors upon reasonable request by suitably qualified investigators.

## Abbreviations

ACE-III: Addenbrooke’s cognitive examination-III
CCR7: C–C chemokine receptor type 7
CD: Cluster of differentiation
CMV: Cytomegalovirus
CNS: Central nervous system
CRP: C-reactive protein
CSF: Cerebrospinal fluid
EBV: Epstein–Barr virus
EDTA: Ethylenediaminetetraacetic acid
FACS: Fluorescence-activated cell sorting
hTERT: Human telomerase reverse transcriptase
LEDD: Levodopa equivalent daily-dose
MDS-UPDRS: MDS-Unified Parkinson's Disease Rating Scale
p16^INK4a^: Cyclin-dependent kinase inhibitor 2A
p21^CIP1/Waf1^: Cyclin-dependent kinase inhibitor 1
PBMCs: Peripheral blood mononuclear cells
PD: Parkinson’s disease
PGK1: Phosphoglycerate kinase 1
PTK7: Protein tyrosine kinase 7
RTE: Recent thymic emigrant
RT-qPCR: Real-time quantitative polymerase chain reaction
TEMRA: Terminally differentiated effector memory cells re-expressing CD45RA
